# Current status and associated factors of social avoidance in postoperative breast cancer patients: a cross-sectional study

**DOI:** 10.3389/fpsyt.2025.1679997

**Published:** 2026-01-12

**Authors:** Xian Chen, Xueli Yang, Qian Zhang, Tianyu Chu, Jiajia Zhang, Linlin Ma, Ling Chen, Jie Zhao

**Affiliations:** 1 Human Reproductive and Genetic Center, Affiliated Hospital of Jiangnan University, Wuxi, China; 2 Department of Breast Surgery, Affiliated Hospital of Jiangnan University, Wuxi, China; 3 Department of Nursing, Affiliated Hospital of Jiangnan University, Wuxi, China

**Keywords:** breast cancer, social avoidance, post-operative, self-disgust, acceptance of disability, nursing

## Abstract

**Background:**

Compared with the general population, breast cancer patients undergoing chemotherapy after surgery are more likely to suffer from multiple psychological problems. Social avoidance, as a negative psychological factor, aggravates anxiety and depression, which in turn reduces patients’ socialization ability and seriously affects their spirituality, mental health, physical health, and ability to cope with the disease. However, research on social avoidance and distress in breast cancer patients remains limited. In light of the growing public concern about these issues, this study aimed to investigate the extent of social avoidance in postoperative breast cancer patients and to explore the factors that influence social avoidance.

**Methods:**

In this cross-sectional study, 185 breast cancer patients were recruited from the Affiliated Hospital of Jiangnan University between December 2024 and May 2025. Participants completed standardized questionnaires measuring social avoidance, self-disgust, and acceptance of disability. Statistical analyses included factor identification through univariate and multivariate models, and correlation assessments to explore relationships between the study variables.

**Results:**

The mean score of social avoidance and distress in 185 postoperative breast cancer patients was (10.75 ± 4.27), of which, social avoidance and social distress scores were (5.24 ± 2.39) and (5.51 ± 2.33), respectively. Educational levels, receipt of postoperative breast cancer education, enlargement of scope of values, containment of disability effect, and behavioural disgust were predictors of social avoidance (*p* < 0.05). Social avoidance was negatively correlated with acceptance of disability (r = -0.585, *p* < 0.01), and acceptance of disability was negatively correlated with self-disgust (r = -0.676, *p* < 0.01). Social avoidance was significantly and positively correlated with self-disgust (r = 0.550, *p* < 0.01).

**Conclusion:**

The study found that 55.1% of breast cancer patients exhibited intermediate levels of social avoidance. Consequently, healthcare professionals are in a position to develop targeted interventions based on these factors to guide patients to tap into positive and active psychology, improve the level of social avoidance, and ultimately improve the quality of life. This, in turn, provides a basis for better implementation of psychological interventions for social avoidance in the future.

## 1 Introduction

According to the latest global estimates from the International Agency for Research on Cancer (IARC), breast cancer (BC) remains the most common cancer among women worldwide, with over 2.3 million new cases reported in 2022 and projections reaching 3.2 million by 2050 (1). In China, BC accounted for 57.4% of new cases among the top 5 most common cancers (2). The advent of screening technologies has led to a marked increase in the detection of early-stage lesions and patient survival rates, attributable to the increased popularity of BC screening (3). Concurrently, extended survival periods have brought the array of psychological and social challenges that patients face after completing treatment into sharper focus. These challenges constitute the ‘psychosocial sequelae’ of the disease. Surgical intervention remains the cornerstone of BC treatment, including breast-conserving surgery, modified radical mastectomy, unilateral/bilateral mastectomy, and breast reconstruction, supplemented by radiotherapy, chemotherapy, and drug therapy (4). The objective of breast-conserving surgery is to excise the tumour while preserving the majority of the breast tissue. In contrast, mastectomy comprises a more extensive range of procedures, including skin-sparing or nipple-areola complex-sparing mastectomy, unilateral or bilateral total mastectomy, and others (5). Notably, in many cases, mastectomy can be performed concurrently with immediate breast reconstruction. Plastic surgeons reshape the breast contour using implants or autologous tissue flaps, helping women achieve a more aesthetically pleasing breast form (6). This has become an integral part of modern comprehensive BC treatment.

However, irrespective of the surgical approach, the procedure itself profoundly impacts patients’ body image (7). The concept of body image is widely understood to encompass an individual’s subjective perception and emotional experience of their own body (8). As a significant symbol of femininity, sensuality, and self-identity, the post-surgical alteration of the breast—whether partial loss, total loss, or the acquisition of a “new” breast through reconstruction—significantly disrupts patients’ self-perception (9, 10). Research indicates that patients undergoing total mastectomy typically experience significantly higher levels of body image distress and body dysmorphism compared to those who undergo breast-conserving surgery (10, 11). Moreover, even though breast reconstruction can effectively improve body image satisfaction, patients may still require a prolonged psychological adjustment period to accept this “new body part” (12). The physical changes resulting from BC surgery and related treatments (e.g. incision scars and chemotherapy side effects) have the potential to significantly disrupt the integrity of a patient’s body image and undermine their positive identification with their feminine traits, leading to a perceived loss of femininity (13, 14). This subjective experience constitutes a key source of psychological distress, potentially leading to negative emotions such as anxiety and depression that impact quality of life and interpersonal relationships (15, 16). Research also indicates that the aforementioned conditions may lead patients to adopt negative coping strategies in social interactions. This may result in patients experiencing diminished self-confidence or a perceived reduction in attractiveness, which can lead to an avoidance of social settings. This phenomenon is referred to as social avoidance behaviour (7, 17, 18).

Social avoidance is defined as an individual’s behavioural tendency to refrain from interacting with others in social situations, frequently accompanied by subjective feelings of distress and discomfort (19, 20). Social avoidance is not merely an abstract psychological concept; it exerts multidimensional and profoundly negative impacts on the real lives of BC patients following surgery. This phenomenon directly undermines their social support systems and severely impedes their successful reintegration into society. Furthermore, the fear of negative judgement from others or stigmatisation of illness has been demonstrated to further exacerbate patients’ feelings of isolation and psychological distress. When this fear compounds with visible physical changes, patients often fall into a vicious cycle of self-isolation to avoid anticipated social rejection (21). Longitudinal studies by scholars such as He further reveal that depressive symptoms are a key risk factor predicting patients’ trajectory into “persistent high social avoidance” (17). This finding suggests that individuals experiencing depressive symptoms are more prone to enduring prolonged periods of severe social isolation. In clinical practice, the timely identification and proactive management of social avoidance are pivotal in disrupting this detrimental cycle and facilitating patients’ comprehensive physical, mental, and social rehabilitation.

Self-disgust is defined as an individual’s persistent or recurrent aversion towards certain aspects of themselves, accompanied by a series of avoidance and rejection behaviours (22). Research indicates that individuals experiencing intense dissatisfaction or aversion towards themselves are more prone to clinical symptoms of depression and anxiety (23–25). In the context of BC patients, the clinical significance of self-disgust is a subject that is garnering increasing attention. A notable instance of this phenomenon is observed in BC patients who undergo postoperative body image changes, a subject that has been extensively documented (26). This aversion has been shown to directly erode an individual’s sense of self-worth, leading to low self-esteem, and to diminish their ability to perceive and utilise existing social support. Collectively, these elements serve to exacerbate the patient’s psychological vulnerability, potentially constituting the underlying psychological motivation for the adoption of social avoidance behaviours. This avoidance strategy is employed to circumvent anticipated social evaluation and interaction pressures, which ultimately severely impedes comprehensive recovery (27, 28).

The concept of disability acceptance is defined as an individual’s recognition and acceptance of their disability status, reflecting a profound understanding of personal worth and resilience against external societal pressures (29). A plethora of studies have indicated that individuals who experience elevated levels of dissatisfaction or excessive criticism with regard to their body image are more likely to hold exaggeratedly negative perceptions of their physical appearance (30). Moreover, these individuals demonstrate a heightened propensity to pursue an idealised aesthetic, thereby significantly exacerbating their social appearance anxiety (31). Conversely, higher levels of acceptance of disability have been shown to facilitate patients’ reconstruction of their self-worth, thereby reducing excessive preoccupation with appearance and effectively buffering the negative impact of body image on social appearance anxiety (32, 33). In this process, disability acceptance is considered a key underlying moderating variable. It can be reasonably inferred that a higher level of acceptance of disability may effectively serve as a psychological buffer against social anxiety triggered by concerns regarding body image. This is achieved by helping patients to differentiate between their self-worth and their impaired body image, thereby reducing the likelihood of adopting avoidance behaviours as a psychological defence strategy.

A growing body of research suggests that social avoidance is becoming more prevalent in contemporary society and that this phenomenon, if left unaddressed, which in turn increases the risk of depression and anxiety disorders (17, 18, 21, 34). In addition, negative self-evaluations can hinder an individual’s ability to effectively cope with life stressors and challenges, further exacerbating their psychological distress. Among the psychological challenges faced by individuals with BC, social avoidance, self-disgust, and disability acceptance are three key factors that can affect an individual’s mental health, quality of life, and overall recovery. However, extant research on postoperative psychological adaptation in BC patients has primarily focused on psychological distress such as anxiety and depression, with limited in-depth exploration of social avoidance—a specific behaviour that significantly impacts social functioning. In particular, it remains unclear how self-disgust and acceptance of disability, two potentially key underlying psychological mechanisms, influence the development of social avoidance. The present study aims to systematically map the current state of social avoidance among postoperative BC patients, with a particular focus on examining the roles of self-disgust and disability acceptance. This also provides a theoretical foundation for prospective positive psychological interventions for patients.

## 2 Materials and methods

### 2.1 Participants

A convenience sampling method was employed to select 185 BC patients who had undergone surgical treatment at the Breast Surgery Department of the Affiliated Hospital of Jiangnan University in Wuxi, China, from November 2024 to May 2025 for the study. The inclusion criteria for the study are as follows: First, subjects aged ≥18 years; Second, female patients with a pathologically confirmed diagnosis of BC; Third, patients who had undergone breast surgery; Fourth, subjects were conscious, capable of writing, free from cognitive impairment, psychiatric disorders, or communication barriers, and provided informed consent to voluntarily participate in the study. The following criteria serve as exclusionary factors: First, a documented history of mental illness or cognitive impairment; Second, the presence of other serious acute or chronic diseases; Third, the presence of other tumours. The study was conducted in accordance with the ethical guidelines established by the Declaration of Helsinki and received approval from the Ethics Committee of the Affiliated Hospital of Jiangnan University (approval number: JSMS04240762). All enrolled patients provided written informed consent.

The present study employed G*Power 3.1 software for the estimation of sample size, setting the significance level (α) at 0.05 and the statistical power (1-β) at 0.80 (35). In order to examine the combined effects of multiple predictor variables on social avoidance, a linear multiple regression model was selected as the computational basis. The anticipated effect size was determined to be small to moderate (f² = 0.10), a conclusion that was supported by the findings of earlier comparable studies. It is evident from the calculations that a minimum of 150 participants is required to detect this effect size. It is estimated that a 90% response rate to the questionnaire will be achieved, and 167 patients are to be recruited to ensure that the final sample size meets statistical requirements.

### 2.2 Measures

#### 2.2.1 The demographic and clinical questionnaire

Researchers employed a self-designed general condition questionnaire for the survey, covering both general demographic information and disease-related details. Its content is based on a literature review and was reviewed by two breast surgery specialists and one epidemiology expert to ensure validity. The general information included the following: age; body mass index (BMI); occupation; marital status; years of marriage; education level; monthly family income; religious beliefs; long-term residence; mode of payment for medical expenses; tumour stage; type of surgery; postoperative time; current or previous treatment; whether they had received postoperative education; and whether they had metastasis or recurrence after surgery. The survey scope also encompassed current or prior postoperative treatment modalities (chemotherapy, radiotherapy, endocrine therapy, etc.), postoperative BC education, and postoperative metastasis or recurrence. The ‘Other’ treatment category in the collection of treatment histories is intended to encompass all additional therapies beyond surgery, chemotherapy and radiotherapy. This includes novel treatments such as targeted therapy and immunotherapy, among others.

#### 2.2.2 The Chinese version of social avoidance and distress scale

The scale was developed by Watson and Friend et al. in 1969 (19), and the Chinese version was revised and completed by Ma Hong (36). The scale under consideration consists of 28 items, including two dimensions of social avoidance (14 items) and social distress (14 items). The Cronbach’s alpha of the social avoidance and distress scale was 0.85 for the avoidance subscale and 0.87 for the distress subscale. In this study, the Cronbach’s alpha coefficient of the scale was 0.90, indicating good reliability.

#### 2.2.3 The Chinese version of the questionnaire for the assessment of self-disgust

The scale was developed by Schienlel in 2014 for the purpose of studying self-disgust in patients diagnosed with depression (37). Since then, it has gained widespread popularity and has been adopted by patients suffering from chronic illnesses. The scale comprises 14 items, encompassing both personal disgust (9 items) and behavioural disgust (5 items). The scale was introduced and Chine seized by Jin Yanfei (38), and the Cronbach’s coefficient of the scale was 0.89, and the re-test reliability was 0.81. The Cronbach’s coefficient of the scale in the present study was 0.87, indicating good reliability.

#### 2.2.4 The Chinese version of the acceptance of disability scale

The scale was developed by Linkowski in 1971 based on the theory of loss acceptance (29), and was primarily used to assess individuals’ perception and acceptance of their own physical status disability. The scale was later translated into Chinese by Chen Ni et al. (39), resulting in its widespread use in clinical practice. The scale under consideration consists of four dimensions, including enlargement of scope of values, subordination of physique, containment of disability effect, and transformation from comparative values to asset values, with a total of 32 entries. The reliability of the scale was determined to be 0.83 and 0.92, respectively.

#### 2.2.5 Quality control

A pilot test with 40 patients was conducted prior to the formal survey to identify potential issues. During the formal survey, all participants received standardized instructions, and objective explanations were provided for any unclear items. Questionnaires were collected immediately upon completion and checked for omissions or inconsistencies. After verification, they were systematically numbered and entered into the database. A random sample of 10% of questionnaires was re-checked to ensure data accuracy.

#### 2.2.6 Statistical analyses

Data were analysed using SPSS 25.0 under the guidance of a statistician. Continuous variables were assessed for normality using the Shapiro-Wilk test and Q-Q plots. Normally distributed data are presented as mean ± SD and analysed using parametric tests (t-test, ANOVA); non-normal data as median (IQR) with non-parametric tests (Mann-Whitney, Kruskal-Wallis). Missing data were handled using multiple imputation after confirming missing completely at random pattern. Relationships between key variables (social avoidance, self-disgust, disability acceptance) were examined using Pearson or Spearman correlation based on distribution. Multiple linear regression identified predictors of social avoidance after verifying assumptions: linearity (partial regression plots), homoscedasticity (residual plots), independence of residuals (Durbin-Watson ≈ 2), and absence of multicollinearity (VIF < 10). Statistical significance was set at *p* < 0.05 (two-tailed).

## 3 Results

### 3.1 Socio-demographic characteristics of patients

This study included 185 patients with BC. All were female and aged 22–63 years (50.7 ± 10.7). Of these patients, 58.9% had an educational attainment level of junior high school or below; 54.1% were retired; 52.4% had lived in urban areas for a long time; 37.8% had employee medical insurance; 31.9% had undergone surgery more than a year previously; 98.9% had received BC education after surgery; and 50.8% had experienced postoperative metastasis or recurrence. The detailed sociodemographic characteristics of the participants are presented in **Table 1**.

**Table 1:** General information on BC patients.

Variables	Categories	N=185	Percentage
Age (years)	18-35	21	11.4
	36-50	46	24.9
	51-65	118	63.8
Stage	I	9	4.9
	II	59	31.9
	III	63	34.1
	IV	54	29.2
BMI (kg/m^2^)	<25	116	62.7
	≥25	69	37.3
Educational levels	Primary school and below	44	23.8
	Junior high school	65	35.1
	High school or secondary school	43	23.2
	Junior college	20	10.8
	Undergraduate and above	13	7.0
Marital status	Unmarried	6	3.2
	Married	153	82.7
	Divorced	15	8.1
	Widowed	11	5.9
Length of marriage (years)	<10	18	9.7
	10-30	72	38.9
	>30	95	51.4
Occupation	Employed	65	35.1
	Unemployed	20	10.8
	Retired	100	54.1
Religious beliefs	Yes	53	28.6
	No	132	71.4
Permanent residence	Cities and towns	97	52.4
	Countryside	88	47.6
Average monthly household income (RMB)	<3000	20	10.8
	3001-5000	64	34.6
	5001-10000	67	36.2
	>10000	34	18.4
Type of medical insurance	Self-financed	6	3.2
	Employee medical insurance	70	37.8
	Residents’ medical insurance	49	26.5
	New rural cooperative medical insurance	57	30.8
	Other	3	1.6
Type of surgery	Breast conservation surgery	56	30.3
	Modified radical mastectomy for breast cancer	106	57.3
	Mastectomy (unilateral or bilateral)	23	12.4
Postoperative time (months)	<3	51	27.6
	3-6	43	23.2
	6-12	32	17.3
	>12	59	31.9
Current or previous treatments	Surgery + chemotherapy	152	82.2
	Surgery + radiotherapy	3	1.6
	Surgery + chemotherapy + radiotherapy	20	10.8
	Surgery + chemotherapy + radiotherapy + other	10	5.4
Whether or not they received breast cancer education after surgery	Yes	183	98.9
	No	2	1.1
Whether metastasis or recurrence after surgery	Yes	91	49.2
	No	94	50.8

### 3.2 Scores of social avoidance and distress in postoperative BC patients

The average scores were as follows: Social avoidance and distress: 10.75 (4.27); Self-disgust: 18.95 (12.45); Disability acceptance: 81.86 (16.01). The average scores for the other dimensions are shown in **Table 2**.

**Table 2:** Mean scores of variables and subcategories.

Variables	Entries	M ± SD
Social avoidance and distress	28	10.75 ± 4.27
social avoidance	14	5.24 ± 2.39
social distress	14	5.51 ± 2.33
Self-disgust	14	18.95 ± 12.45
personal disgust	9	12.28 ± 8.06
behavioural disgust	5	6.68 ± 4.63
Acceptance of disability	32	81.86 ± 16.01
enlargement of scope of values	9	24.14 ± 6.86
subordination of physique	9	22.90 ± 5.37
containment of disability effect	5	11.41 ± 2.87
transformation from comparative values to asset values	9	23.38 ± 4.92

### 3.3 Univariate analysis of variance for social avoidance and distress in BC patients

Single-factor analysis of variance revealed significant differences in social avoidance scores based on age, educational level, occupation, permanent residence, medical insurance type, postoperative duration, receipt of BC education post-surgery, and presence of recurrence or metastasis (*p* < 0.05). These factors demonstrated statistically significant variations in social avoidance scores. (see **Table 3**).

**Table 3:** A unifactorial analysis of social avoidance in postoperative BC patients.

Variables	N=185	M ± SD	t/F	*p*
Age (years)			6.495	0.002*
18-35	21	12.29 ± 5.614		
36-50	46	12.17 ± 3.672		
51-65	118	9.92 ± 4.022		
Stage			0.685	0.562
I	9	9.44 ± 3.395		
II	59	11.00 ± 4.064		
III	63	11.10 ± 4.272		
IV	54	10.30 ± 4.624		
BMI (kg/m^2^)			0.196	0.845
<25	116	10.80 ± 3.890		
≥25	69	10.67 ± 4.868		
Educational levels			4.708	0.001*
Primary school and below	44	8.80 ± 3.951		
Junior high school	65	10.88 ± 4.033		
High school or secondary school	43	11.44 ± 4.211		
Junior college	20	11.15 ± 4.308		
Undergraduate and above	13	13.85 ± 4.337		
Marital status			0.766	0.515
Unmarried	6	9.33 ± 3.983		
Married	153	10.82 ± 4.192		
Divorced	15	11.60 ± 5.343		
Widowed	11	9.45 ± 4.009		
Length of marriage (years)			2.962	0.054
<10	18	12.17 ± 4.890		
10-30	72	11.32 ± 4.373		
>30	95	10.05 ± 3.972		
Occupation			4.687	0.010*
Employed	65	12.03 ± 4.500		
Unemployed	20	10.15 ± 4.557		
Retired	100	10.04 ± 3.890		
Religious beliefs			-0.584	0.560
Yes	53	11.00 ± 3.205		
No	132	10.65 ± 4.635		
Permanent residence			5.832	0.017*
Cities and towns	97	11.46 ± 3.865		
Countryside	88	9.97 ± 4.567		
Average monthly household income (RMB)			1.888	0.133
<3000	20	8.95 ± 4.582		
3001-5000	64	10.64 ± 4.952		
5001-10000	67	10.88 ± 3.879		
>10000	34	11.76 ± 3.085		
Type of medical insurance			3.098	0.017*
Self-financed	6	13.50 ± 1.643		
Employee medical insurance	70	11.71 ± 4.569		
Residents’ medical insurance	49	10.65 ± 3.467		
New rural cooperative medical insurance	57	9.40 ± 4.391		
Other	3	10.00 ± 3.606		
Type of surgery			0.509	0.602
Breast conservation surgery	56	10.96 ± 4.828		
Modified radical mastectomy for breast cancer	106	10.50 ± 4.039		
Mastectomy (unilateral or bilateral)	23	11.39 ± 3.928		
Postoperative time (months)			4.109	0.008*
<3	51	12.41 ± 3.864		
3-6	43	10.37 ± 4.826		
6-12	32	10.56 ± 4.662		
>12	59	9.69 ± 3.573		
Current or previous treatments			0.488	0.691
Surgery + chemotherapy	152	10.66 ± 4.503		
Surgery + radiotherapy	3	11.00 ± 1.000		
Surgery + chemotherapy + radiotherapy	20	11.75 ± 3.275		
Surgery + chemotherapy + radiotherapy + other	10	10.00 ± 2.539		
Whether or not they received breast cancer education after surgery			25.159	<0.001*
Yes	183	10.84 ± 4.213		
No	2	3.00 ± 0.000		
Whether metastasis or recurrence after surgery			-2.566	0.011*
Yes	91	9.95 ± 4.215		
No	94	11.53 ± 4.196		

*Significant at *p* < 0.05 level.

### 3.4 The correlations between social avoidance, acceptance of disability, and self-disgust

Pearson correlation analyses were conducted to examine the relationships between social avoidance, acceptance of disability and self-disgust. The results showed that social avoidance was negatively correlated with disability acceptance (r = −0.585, *p* < 0.01) and positively correlated with self-disgust (r = 0.550, *p* < 0.01). Additionally, disability acceptance was found to be negatively correlated with self-disgust (r = −.676, *p* <.01). The full correlation matrix is presented in **Table 4**.

**Table 4:** Correlations between social avoidance and distress, self-disgust, and acceptance of disability.

Variables	SAD	Social avoidance	Social distress	Acceptance of disability
Acceptance of disability	-0.585**	-0.525**	-0.531**	**-**
Enlargement of scope of values	-0.445**	-0.398**	-0.407**	0.739**
Subordination of physique	-0.527**	-0.471**	-0.480**	0.896**
Containment of disability effect	-0.324**	-0.293**	-0.293**	0.506**
Transformation from comparative values to asset values	-0.517**	-0.470**	-0.464**	0.944**
Self-disgust	0.550**	0.542**	0.450**	-0.676**
Personal disgust	0.527**	0.523**	0.428**	-0.672**
Behavioural disgust	0.561**	0.546**	0.466**	-0.648**

SAD, social avoidance and distress.

*Significant at *p* < 0.05 level.

**Significant at *p* < 0.01 level.

### 3.5 Multiple linear regression analysis of factors influencing social avoidance in postoperative BC patients

A multivariate linear regression analysis was conducted using the statistically significant variables from the univariate analysis as independent variables and social avoidance and distress as dependent variables. Results indicated a significant regression model, F (11, 173) = 13.70, *p* < 0.001, explaining 49.1% of the variance in social avoidance (adjusted R² = 0.491). Significant predictors included age, education level, enlargement of scope of values, containment of disability effects, and behavioural disgust. Detailed regression coefficients are presented in **Table 5**.

**Table 5:** Multiple linear regression analysis of factors influencing social avoidance and distress in BC patients.

Category	B	SE	Beta	t	*p*
Constant term	25.947	3.827	–	6.779	<0.001*
Enlargement of scope of values	-0.126	0.056	-0.202	-2.258	0.025*
Subordination of physique	-0.088	0.096	-0.111	-0.917	0.360
Containment of disability effect	-0.287	0.121	-0.193	-2.380	0.018*
Transformation from comparative values to asset values	-0.021	0.110	-0.024	-0.191	0.849
Personal disgust	-0.073	0.075	-0.139	-0.976	0.330
Behavioural disgust	0.393	0.126	0.426	3.108	0.002*
Age	0.716	0.527	0.116	1.359	0.176
Educational levels	0.756	0.284	0.207	2.658	0.009*
Occupation	-0.420	0.281	–0.091	-1.495	0.137
Permanent residence	-0.658	0.568	-0.077	-1.157	0.249
Type of medical insurance	-0.080	0.321	-0.018	-0.250	0.803
Postoperative time	-0.330	0.213	-0.093	-1.551	0.123
Whether or not they received breast cancer education after surgery	-9.368	2.294	-0.228	-4.084	<0.001*
Whether metastasis or recurrence after surgery	0.461	0.510	0.054	0.903	0.368

R^2^ = 0.530, Adjusted R^2^ = 0.491, F = 13.700, *P* < 0.001.

*Significant at *p* < 0.05 level.

## 4 Discussion

This study confirms that BC patients undergoing postoperative treatment often exhibit varying degrees of social avoidance, with more than half reporting moderate to severe avoidance behaviours. This finding highlights the importance of addressing patients’ psychosocial adaptation capabilities and implementing targeted interventions in BC clinical care. Restoring social functioning is a crucial indicator of recovery and a means for patients to rebuild their social identity and gain emotional support. This directly impacts patients’ ability to cope with the disease and their long-term quality of life, offering significant insights into how to enhance the social functioning of postoperative BC patients (40).

Contrary to the findings of some previous studies, this study found a positive correlation between educational attainment and social avoidance, which is consistent with the findings of Li et al. (32). One possible explanation for this apparent paradox is the ‘fear of status loss’. Patients with higher levels of education tend to occupy more prominent roles within professional and social networks. Postoperative changes in body image can trigger deep-seated anxiety about losing social status and self-worth (41, 42). In order to protect their sense of self-worth, they may proactively choose to withdraw from social situations as a risk-avoidance strategy against the potential threat of social evaluation (21, 43). This contrasts with the pathway identified in Kuang et al.’s study, in which patients with lower levels of education exhibited social withdrawal due to insufficient knowledge of their condition and inadequate self-management capabilities (44). This highlights the complexity of how educational background influences patient behavioural patterns through distinct psychosocial mechanisms.

Of particular note, patients who received health education exhibited higher levels of social avoidance. This counterintuitive finding may be indicative of individual differences in health information processing. For some patients who are psychologically vulnerable, intensive disease knowledge education, particularly regarding recurrence risks and physical sequelae, may exceed their immediate coping capacity. This may result in exacerbation of catastrophic thinking about the disease and fear of social situations (45). Concurrently, “selective bias” in clinical practice may also contribute to this phenomenon, as healthcare providers tend to deliver more intensive and frequent health education to patients exhibiting higher anxiety levels or more complex prognoses (46). The observed association may be partly indicative of pre-existing psychological risk in this group, rather than education itself leading to avoidance behaviour. This finding indicates that future health education initiatives should extend beyond the mere transmission of knowledge, incorporating psychological assessment and support, and personalising content based on patients’ psychological readiness (25).

The present study indicates that acceptance of disability serves as a key protective factor against social avoidance. Individuals who exhibit high levels of disability acceptance have been found to demonstrate a greater capacity for proactive adjustment of their self-perception and adaptation to physical changes, thereby leading to a reduction in social avoidance (47, 48). This phenomenon is inextricably linked to the vital role of family and social support. A robust support network has been shown to provide resources for rehabilitation and to promote patients’ positive acceptance of disability (49). Consequently, clinical interventions should concentrate on establishing supportive environments that encourage patients to proactively seek and effectively utilise social resources. Furthermore, the presence of self-loathing, as the core emotional component of body image distress, has been demonstrated to exhibit a significant positive correlation with social avoidance. Within the cognitive-behavioural framework, self-loathing is defined as a direct emotional response to perceived bodily defects, with avoidance being its core behavioural manifestation (48).

In order to alleviate social avoidance behaviours in BC patients post-surgery, clinical interventions should adopt multidimensional strategies. It is recommended that healthcare providers incorporate psychological assessments into routine care, deliver personalised health education based on patients’ psychological readiness and educational background, and emphasise the cultivation of social coping skills. Family members and friends must be educated in the provision of non-judgemental emotional support, with the objective of assisting patients in the reconstruction of a positive self-image. Concurrently, patients should be encouraged to proactively utilise support resources, gradually engaging in social interactions within safe, controlled environments. The integration of psychological support with cognitive-behavioural techniques has been demonstrated to be an effective strategy for the regulation of self-disgust and the enhancement of disability acceptance. This, in turn, can facilitate the establishment of positive social connections and promote long-term psychosocial adaptation in patients.

## 5 Limitations

Nevertheless, the present study is not without its limitations. First, while cross-sectional survey design is capable of revealing associations between variables, it is not equipped to infer causal direction or dynamic evolution, which complicates the clarification of potential causal pathways between variables. Secondly, the data were collected exclusively through self-administered questionnaires. Despite the demonstrated reliability and validity of the instruments, it remains unfeasible to entirely preclude the potential influence of common method bias and social desirability bias on the magnitude of variable associations. A third limitation is the restriction of the generalizability of the findings due to the origin of all samples from a single centre: the Affiliated Hospital of Jiangnan University. As a regional medical institution, its diagnostic protocols, nursing resources, and postoperative management plans may differ from those in other regions or countries. The findings are significantly impacted by the local sociocultural context, and the relative homogeneity of the sample’s demographic characteristics compromises its representativeness for more extensive populations of postoperative BC patients. This study employed clinical staging rather than TNM staging for grouping. While this approach aligns with the study’s sociopsychological focus and ensures statistical power, future research with larger sample sizes may explore associations between more refined TNM subgroups and psychosocial outcomes. Additionally, in the treatment history survey, we categorised novel ‘smart drugs’, such as targeted therapy and immunotherapy, under the ‘other’ option. This limited our ability to conduct independent analyses of the efficacy of these specific therapies. Future studies should use more detailed classifications of treatment regimens. The subsequent step should entail an augmentation of the sample size through the integration of longitudinal and cross-sectional qualitative research, with the objective of exploring the long-term mechanisms and developmental trajectories of social avoidance behaviour. It is recommended that future multicentre studies encompass broader geographic regions and diverse healthcare settings in order to validate the external validity of research findings.

## 6 Conclusion

Overall, this study found that postoperative BC patients exhibited moderate levels of social avoidance. Additionally, the study revealed that social avoidance, distress, disability acceptance, and self-disgust were closely related among patients. Our study reveals that significant mental health problems, such as social avoidance and self-disgust, are prevalent among postoperative BC patients to varying degrees. Therefore, relevant organizations must urgently address these issues, especially among patients with lower education, low perceived self-worth, and low disability acceptance. Healthcare professionals can develop interventions tailored to each patient to enhance self-awareness and acceptance of disability, thereby improving social functioning. These comprehensive enhancements are essential for guiding patients to cope with the challenges of the disease positively, improving quality of life, and promoting full recovery. Furthermore, healthcare professionals can be trained in brief, structured, supportive conversation techniques, utilising motivational interviewing to encourage patients to engage in social activities and seek professional psychological support. The implementation of targeted strategies is imperative for the effective alleviation of social avoidance, the promotion of psychological adaptation, and the ultimate enhancement of the long-term quality of life for this demographic.

## Data Availability

The original contributions presented in the study are included in the article/supplementary material. Further inquiries can be directed to the corresponding authors.
